# From Notebooks to Institutions: The Case for Symbiotic Cognition

**DOI:** 10.3389/fpsyg.2020.00674

**Published:** 2020-04-09

**Authors:** Marc Slors

**Affiliations:** Faculty of Philosophy, Theology and Religious Studies, Radboud University, Nijmegen, Netherlands

**Keywords:** extended cognition, socially extended cognition, cognitive integration, distributed cognition, symbiotic cognition

## Abstract

Cognition is claimed to be extended by a wide array of items, ranging from notebooks to social institutions. Although the connection between individuals and these items is usually referred to as “coupling,” the difference between notebooks and social institutions is so vast that the meaning of “coupling” is bound to be different in each of these cases. In this paper I argue that the radical difference between “artifact-extended cognition” and “socially extended cognition” is not sufficiently highlighted in the literature. I argue that there are two different senses of “cognitive extension” at play, that I shall label, respectively, “implementation extension” and “impact extension.” Whereas implementation extension is a causal-functional notion, impact-extension hinges on social normativity that is connected with organization and action coordination. I will argue that the two kinds of cognitive extension are different enough to warrant separate labels. Because the most salient form of social extension of cognition involves the reciprocal co-constitution of cognitive capacities, I will propose to set it apart from other types of extended cognition by using the label “symbiotic cognition.”

## Introduction

In the literature on extended, integrated and distributed cognition, human cognitive systems are said to be coupled with and enhanced by a large number of rather diverse items, ranging from simple notebooks and abacuses (Clark and Chalmers, 1998), via complete physical environments such as theater set-ups (Tribble, 2005; Clark, 2008; Sutton, 2010) to language (Clark, 2008) and social institutions such as legal systems (Fuchs and De Jaegher, 2009; Gallagher and Crisafi, 2009; De Jaegher et al., 2010; Gallagher, 2013; Gallagher et al., 2019). This range is so wide, and the difference between e.g., a notebook and a social institution so immense, that it seems unlikely that people are connected with these items in basically the same way. In this respect it doesn’t matter whether we speak of cognitive integration (Menary, 2007, 2010), “distributed cognition” (Hutchins, 1995; Hutto and Myin, 2017) or of cognitive extension (Clark and Chalmers, 1998; Clark, 2008; Gallagher, 2013). The point is that just saying that we are “functionally integrated” (Heersmink, 2015) with items or “causally coupled” with them is bound to sweep an important difference under the carpet when these items are so radically different. The aim of this paper is to characterize the difference between the way our cognition is extended by and/or integrated with items such as notebooks, abacuses, and smart phones on the one hand—which I will call artifact-extended cognition—and items such as social institutions, language, and cultural conventions—which is known as socially extended cognition—on the other. I will argue that the difference is significant enough for the latter kind of extension/integration to warrants its own separate label, for which I will propose the term “symbiotic cognition.” In order not to complicate the discussion unnecessarily, I will concentrate on the literature on extended cognition, except for the last section of this paper.

The paper is set-up as follows. In the next section I will introduce the notion of extended cognition and highlight the difference between artifact-extended cognition and socially extended cognition. In the section “The Problem of Cognitive Bloat,” I will briefly discuss the problem of cognitive bloat such as this has first been proposed as an argument against the early varieties of cognitive extension. I will argue that if socially extended cognition is indeed modeled on artifact-extended cognition, it falls prey to this problem in such a blatant way that it is clear that we must understand socially extended cognition differently. In the section “Implementation-Extension and Impact-Extension,” I will propose a characterization of the difference between artifact-extended cognition and socially extended cognition. I will argue that cognition can be considered to be extended in different ways. Whereas artifact-extended cognition extends cognitive processes by extending the *implementation base* of these processes, socially extended cognition alters the nature and hence extends the *impact* of cognitive engagements with the world by embedding them in social practices of coordinated behavior. When we interpret socially extended cognition as an instance of impact-extension and not as implementation-extension, the problem of cognitive bloat disappears.

In the section “Causality, Coordination, and Reciprocal Cognitive Dependency,” I will defend and elaborate on the distinction between “implementation-extension” and “impact-extension” by arguing that, crucially, the chain of items causally linked to a person whose cognition is socially extended involves other human beings—other cognitive systems. On the one hand, this introduces social normativity into the extended system, which is absent in artifact-extended cognition. On the other it introduces the idea of reciprocal cognitive dependency between people. I will propose the label “symbiotic cognition” for networks of mutually dependent cognitive systems. In the section “Cognitive Symbiosis, Weak and Strong,” I will define the notion of symbiotic cognition. I will allow for the possibility of socially extended cognition that is not symbiotic cognition, and will distinguish between weak forms of symbiotic cognition, that do not require social institutions, and strong forms that do. In the section “Symbiotic Cognition, Cognitive Integration and Distributed Cognition,” I will compare the idea of symbiotic cognition with integrated cognition (Menary, 2007, 2010, 2013) and distributed cognition (Hutchins, 1995; Hutto and Myin, 2017). I will argue that although some elements of symbiotic cognition surface in these views, the essential contrast between artifact- and social extension is still ignored by both.

## Artifact-Extended and Socially Extended Cognition

The idea that human cognitive systems are in fact extended by items outside our brains and bodies has been developed and defended by many philosophers for over two decades now. Disregarding precursors, the idea that started the debate on extended cognition—then labeled “active externalism” (Hurley, 2010)—was based on the so-called parity principle: “If, as we confront some task, a part of the world functions as a process which were it done in the head, we would have no hesitation in recognizing as part of the cognitive process, then that part of the world is (…) part of the cognitive process.” (Clark and Chalmers, 1998, 8) The (in)famous and widely discussed Otto and Inga example exemplifies this principle: Inga wants to visit the MoMa in New York and remembers that it is on 11 West 53rd Street. Otto has early onset Alzheimer. Instead of relying on information storage in his head, he uses a notebook that he always carries with him. When he wants to visit the MoMa he consults his notebook to find the address. Because the system consisting of Otto-and-notebook is functionally equivalent to Inga, Clark and Chalmers claim that the notebook is as much part of Otto’s mind as the memory storage region of Inga’s brain is part of hers.

Brain-chauvinists think there is a relevant difference. On their view, Otto does not remember the MoMa address. Rather, he believes the address is in the notebook, perceives the contents of the notebook and forms a new belief about the address. On this reconstruction all the mental work is done in Otto’s head, not outside it. The response to this “Otto two-step” (Clark, 2010, 46) is easy enough to imagine: if Otto believes the required information is stored in the notebook and retrieved by perceiving the notebooks contents, then why not say that Inga believes the information she seeks is stored in her brain and she introspects the retrieval of that information, forming a new belief about the address of the MoMa? If we think this reconstruction of Inga’s remembering is contrived then why is a similar reconstruction of Otto’s mental processing not also contrived? The point is that the Otto two-step works only if we are already inclined toward brain-chauvinism.

Some philosophers have argued that we have reason to be chauvinist (Adams and Aizawa, 2001, 2008, 2010). These reasons involve an appeal to “non-derived mental content.” But that is a controversial notion (Dennett, 1978, 1987; Clark, 2010; Hutto and Myin, 2013, 2017). I shall not go into the debate on non-derived content, because it is associated mostly with the “first-wave” extended cognition theories based on the parity principle.^1^ The wider variety of items that our cognition is said to be coupled with, which is the main topic of this paper, stems mainly from the second wave of extended mind theories. These are not based on the parity principle, but on the complementarity principle (Sutton, 2010): items external to our brains and bodies can contribute to cognition, not because they structurally resemble processes that also occur inside the brain, but because they complement brain processes and by doing so allow for new cognitive possibilities. With the first-wave, parity principle-based extended cognition, it is possible to ask whether an extended process that resembles a brain process is as good as “the real thing” (and those who believe in non-derived content think it isn’t). With the second-wave, complementarity principle-based type of extended cognition, this issue does not arise if the cognitive capacities that emerge when an embodied brain is coupled with external devices does not have a merely brain-based equivalent. The issue in such cases is simply whether we are or should be inclined to think of the emerging capacity as a cognitive one.

A prominent example of second-wave extended cognition is the idea that external symbol systems such as written language and numbers extend our cognitive capacities. In Menary’s words “[t]he surrounding linguistic environment contains reliable structures, speech and text, that are available as cognitive resources to be coupled with. Our ability to reliably couple with this ever-present environment constitutes human cognition and thought” (Menary, 2010, 8). Clark agrees by emphasizing that “linguistic tools enable us to deliberatively and systematically sculpt and modify our own processes of selective attention.” (Clark, 2008, 48) Physical symbols, whether written or spoken, bring about capacities for thought, communication and numeracy that are literally unthinkable without them.

The example of language and number-symbols systems is a good stepping stone to the idea of socially extended cognition (Fuchs and De Jaegher, 2009; Gallagher and Crisafi, 2009; De Jaegher et al., 2010; Gallagher, 2013; Gallagher et al., 2019). Just like written language and numbers can extend our cognitive range, the idea behind this position is that some social institutions can do that just as well. I will focus on Shaun Gallagher’s exposition and defense of this idea, because it is the most elaborate version available. Gallagher argues for “a liberal, and specifically social extension of the extended mind hypothesis.” He “appeal[s] to social practices and institutions that are what we might call “mental institutions” (Gallagher and Crisafi, 2009), in the sense that they are not only institutions with which we accomplish certain cognitive processes, but also are such that without them such cognitive processes would no longer exist.” (Gallagher, 2013, p. 6) Examples he uses include legal systems, educational systems and museums, cultural conventions and even the market economy (Gallagher et al., 2019). The idea is that such institutions extend our cognitive capabilities considerably and that this should count as a form of extended cognition.

Our legal system, for example, enables an array of thoughts and actions that would not merely be impossible, but would not even be intelligible without the concept and procedural routines associated with the law. A helpful example that is often used by Gallagher is the practice of formalizing an agreement between two people by signing a contract:

A contract or legal agreement (…) is in some real sense an expression of several minds externalized and extended into the world, instantiating in external memory an agreed-upon decision, adding to a system of rights and laws that transcend the particularities of any individual’s mind. Contracts are institutions that embody conceptual schemas that, in turn, contribute to and shape our cognitive processes (Gallagher, 2013, p. 6).

The point I wish to make in this section is that somewhere along the way in ascending from notebooks as possible cognitive extensions to socio-cultural institutions, a crucial distinction is ignored. The connection between individuals and the items their cognition is extended with is described as more or less similar—it is described as “coupling.” What coupling entails must depend on what we couple with. Hence, in order to maintain similarity throughout the ascent from notebooks to institutions, items that are said to extend our cognition are very often (but not always) described as physical objects. Language, for example, is described as a set of physical symbols. But apart from involving a set of physical symbols, language is also a social *practice*. It is not just scribbles and sounds, but also the way we use these in social interactions.

This certainly goes for social institutions too. Legal systems involve courtrooms, togas, and in some countries wigs. But they also involve rules, conventions and practices. A contract is an externalized memory not just because of its physical properties but mainly because of the way these pieces of paper (or bunches of bits) function in legal practice. Gallagher acknowledges that cognition can also be extended by institutions that are less formal and reinforced, such as practices involving cultural conventions:

In solving a problem like keeping my cattle in my pasture, my bodily manipulations of a set of wooden poles and wire are not necessarily part of the cognitive process; but my engagement with the particular local custom/practice of solving this problem with a fence (and even a specific kind of fence) is a cognitive part of the problem solving. In such cases, cultural practices, local know-how in the form of established practices, etc., in either formal or informal ways, enter into and shape the thinking process. Without such cultural practices, rules, norms, etc. our thinking – our cognitive processes – would be different (Gallagher, 2013, p. 10).

Interestingly, the difference between physical objects and practices is not seen as an obstacle for claiming that coupling is basically similar when we move from notebooks to institutions:

Just as a notebook or a hand-held piece of technology may be viewed as affording a way to enhance or extend our mental possibilities, so our encounters with others, especially in the context of various institutional procedures and social practices may offer structures that support and extend our cognitive abilities (Gallagher, 2013, p.4).

Let us call cognition that is extended by physical objects “artifact-extended cognition.” The (admittedly rhetorical) question I would like to pose is whether Gallagher, Clark and Menary are correct (on some interpretations of their views) in assuming that socially extended cognition is really continuous with artifact-extended cognition. Are coupling with artifacts and coupling with practices really similar enough to warrant the use of the same label—extended cognition—in both instances?

## The Problem of Cognitive Bloat

In order to make a beginning with driving a wedge between artifact-extended cognition and socially extended cognition, it is useful to look at what is known as the problem of cognitive bloat (Rupert, 2004). This is the problem that if we allow notebooks and smart phones to co-constitute our cognitive processes, we may have to include many other things too, in which case we are likely to end up with cognitive processes that are so wide and scattered that it is counterintuitive to think of them as processes of a single person. I will argue that this problem is not alike for artifact-extended cognition and socially extended cognition.

From the perspective of artifact-extended cognition, Otto-and-notebook-style, the response to the threat of cognitive bloat is to tighten the constraints on what counts as co-constituents of cognition. Clark proposes four extra constraints:

(1) That the resource be reliably available and typically invoked. (Otto always carries the notebook and won’t answer that he “doesn’t know” until after he has consulted it).
(2) That any information thus retrieved be more or less automatically endorsed. It should not usually be subject to critical scrutiny (e.g., unlike the opinions of other people). It should be deemed about as trustworthy as something retrieved clearly from biological memory.
(3) That information contained in the resource should be easily accessible as and when required.
(4) That the information in the notebook has been consciously endorsed at some point in the past and indeed is there as a consequence of this endorsement (Clark, 2008, 79).

This does limit the possible candidate artifacts that may be said to extend cognition considerably. Arguably, the remaining problem is a matter of intuition. It is surely the case that even with these extra criteria our extended minds are bigger and more scattered than traditional brain-based or neo-Cartesian intuitions would make them out to be. But they are not so large and scattered that it is incoherent to think of them as single cognitive systems.

One of the reasons for this is that the external items we are said to be coupled with are not themselves coupled with still further structures in ways that satisfy 1–4. But this is exactly the problem with socially extended cognition. If we are coupled with social institutions, we are coupled with structures that are constituted, among other things, by (very many) other human beings. These human beings are themselves coupled with further structures in the same way we are coupled with them. And this makes the cognitive system implausibly large and scattered—if we are able to draw boundaries at all. For this reason, even philosophers who are sympathetic to the idea that human cognition involves massive coupling with our external niches are reluctant to think of social institutions as co-constituents of our cognitive systems (Huebner, 2013; Menary, 2013). According to them it is much more plausible to think of social institutions as the enabling conditions for cognitive abilities such as being able to sign contracts, speaking a language, or using cultural conventions.

The point I wish to make here is not that socially extended cognition clearly falls prey to the problem of cognitive bloat. Rather, the point is that (i) it would fall prey to the problem of cognitive bloat if socially extended cognition is a proposal that is modeled completely on the idea of artifact-extended cognition, and (ii) if it is interpreted in this way it falls prey to the problem of cognitive bloat so obviously and blatantly that it seems unlikely that socially extended cognition is intended to be modeled completely on artifact-extended cognition.

Gallagher is ambivalent here. On the one hand he does present socially extended cognition as a proposal that is somehow derived from the idea of artifact-extended cognition (see the last quote of the previous section “Artifact-Extended and Socially Extended Cognition”). On the other hand, however, he distances himself from Clark’s functionalism and the way Clark deals with the problem of cognitive bloat. Tightening the restrictions on what counts as proper cognitive extension in the way Clark does, emphasizes the idea that the brain is still the central hub of any cognitive system, however extended this system is. And it is precisely such brain-centeredness that Gallagher wishes to overcome with the idea of socially extended cognition. But now the question arises: how is avoiding brain-centeredness and including social practices and institutions in the list of co-constituents of our cognitive processes going to help sidestep the problem of cognitive bloat?

I believe the answer here is to distance the idea of socially extended cognition even more from the idea of artifact-extended cognition than Gallagher does.

## Implementation-Extension and Impact-Extension

To say that cognition is extended is to say that items external to our brains and bodies expand our cognitive repertoire in such a way that they can somehow be said to co-constitute the “mechanisms”^2^ of the cognitive system responsible for that repertoire. Differently put: some of the cognitive work in our interactions with the world has to be performed by items external to our brains and bodies. I believe there are different ways in which these descriptions can be made more precise. And I believe that the way in which we do this depends on our views of what cognition consists of. In this section I will sketch two different ways of unpacking the idea of cognitive extension. One is tailor-made for the functionalist view of cognition that underlies Clark-style artifact-extended cognition. The other is more suitable for Gallagher-style enactivist views of cognition—even though I am less sure he would accept it.

The meaning of cognitive extension that fits a functionalist outlook on cognition such as Clark’s best is what I will label “implementation extension.” According to functionalists, cognitive states and processes are to be characterized as functional role states and transitions from one set of functional states to another [this formulate is an attempt to cover as many variants of functionalism as possible, but at any rate machine functionalism (Putnam, 1967), psycho-functionalism (Fodor, 1968), and analytical functionalism (Lewis, 1972)]. That is, a mental process such as remembering is to be characterized in terms of the function it fulfils for an organism: storage and retrieval of information for the purpose of action control. Functional role states and functional processes are implemented or realized by physical structures that play the appropriate causal roles. Usually these are brain states and processes. The basic idea behind functionalism is that functional role states and processes are multiply realizable: the same function can be physically realized in different ways. And it is precisely this multiple realizability that is put to use in cases such as Otto and Inga, where the same functional process has two different realizations or implementations, one involving brain processes only, the other involving an item in the external world as well. Implementation extension is the idea that the realization or implementation base of functional role states and processes that are characteristic of human cognition includes items outside the brains and bodies of persons.

The notion of implementation extension is probably the most straightforward interpretation of extended cognition, so I will be brief about it. Two things are important to note. First, implementation extension fits really well with artifact extension, since physical artifacts are easy to imagine to be causally coupled with brains and bodies in ways that extend the implementation base of functional processes. It may fit with social extension as well, but as we have seen above this soon leads to an implementation base of extended cognitive processes that covers more than can possibly be said to belong to the cognition of a single person. Secondly, as second-wave extended cognition theories stress, extending the implementation base of functional processes may lead to new functional processes that have no mere brain-based parallel.

The second interpretation of extended cognition is less well-entrenched in philosophy of mind. It may be made compatible with a functionalist outlook, but is fits best with an enactivist notion of cognition. Briefly put, according to enactivists, cognition is a specific type of bodily engagement of an organism with the world [I will, again, try to formulate so as to cover most varieties of enactivism, including autopoietic (Thompson, 2007), sensory-motor (Noë, 2004), and radical (Hutto and Myin, 2013) enactivism]. Cognition is not a hidden layer between perception and action where the real thinking occurs. It is responding to the action-opportunities offered by the environment to an organism in such a way that the organism benefits, e.g., by sustaining its own organization. Cognition is a process that encompasses perception, action and bits of the world. A cognitive process is a specific type of interaction between an organism and the world. Extending a cognitive process in this sense is not extending a realization base of a functional role (because there is no such thing according to enactivists), it is extending the part of the world we can engage with. Differently put, it is increasing the *impact* that a cognitive engagement with the world has, for example on the further action possibilities offered by the environment to the acting organism. Extending the impact of engagements can be achieved by involving specific artifacts in the interaction, but it can also—crucially—be achieved by embedding the interaction in specific social practices.

Some examples of the way in which social practices or institutions extend the impact of cognitive engagements with the world may help to get the idea across. The example of fencing off a piece of land is a good case in point. This relatively simple engagement with the world has the much wider impact of avoiding trespassers on your land only because it is embedded in a context of cultural conventions. But here the impact-extension is still relatively modest. Compare, for example, the process of signing a contract. This is, again, a relatively simple action. But given the legal system in which it is embedded—a system of rules and a practice of using and reinforcing them—as a cognitive engagement it can have a very wide impact. It will change the rights and obligations of the signers, making them house-owners, companions in a firm, employees, etcetera. Or think of a voting process in the board of a large company on a possible reorganization. With five votes for and five against, your vote is the last. By simply raising a hand, you set in motion a large reorganization. Raising a hand is a very modest engagement with the world. But by embedding it in complex of social practices—cultural conventions, economic processes, and legal transactions—its impact is massively extended^3^.

Implementation extension and impact extension are very different forms of cognitive extension—or so I will argue. Many cases of implementation extension start with a pre-existing brain-based cognitive process that is extended by adding external items to the implementation base of these processes. Otto and his notebook are the perfect case in point. Impact extension, specifically if this is social extension, by contrast, involves the creation of new cognitive processes that match pre-existing social practices. As Gallagher rightly stresses, socially extended processes such as signing contracts or voting are not even intelligible in abstraction from the social practices they are part of (see section “Cognitive Symbiosis, Weak and Strong”). Raising a hand or making a scribble on a piece of paper are not cognitive processes at all in abstraction from the relevant social practices that make these engagements instances of voting and signing a contract.

The fact that in “socially impact extended cognition” social practices precede the development of cognitive abilities that help individuals use *and* contribute to these practices suggests a completely different sense in which items outside our brains and bodies can be said to co-constitute our cognitive processes. This is not the type of constitution that is characteristic of the functionalist outlook, where constitution is explained in terms of realization or implementation. Rather than saying that a cognitive process—characterized in functional terms—is constituted by the physical structures that have the relevant causal-functional characteristics, the point here is that certain engagements with the world are parts of the collective behavioral patterns that instantiate a specific social practice. The context of such a practice is needed for these engagements to make them into what they are; to make raising a hand voting and scribbling on a piece of paper signing a contract. In fact, it is more natural to say that these engagements contribute to the perpetuation of institutional practices than it is to say that these practices extend these engagements—though it ultimately boils down to the same claim. It is exactly the fact that these engagements are contributions to institutional practices that explains their (hugely) extended impact, and this makes institutions *co-constitute* these engagements *as* the cognitive processes they are—voting and signing a contract.

I believe that this explanation of what it means to say that social institutions co-constitute some of our cognitive processes is more informative and better applicable to the idea of socially extended cognition than the definition of constitution referred to by Gallagher, 2013 himself in a footnote (2013, 6). According to that definition, “P is a constitutive element [of X] if P is part of the processes that produces X” (De Jaegher et al., 2010, 443). The problem with this definition is that it implies that “[t]he set of all the constitutive elements is the phenomenon itself” (De Jaegher et al., 2010, 443). The suggested identity relation is problematic, since identity is symmetric. But when an act of signing a contract is co-constituted by a legal system, “the set of constitutive elements” is vastly more encompassing than “the phenomenon itself.” A definition of constitution in terms of parts that jointly make up a phenomenon fits better with implementation extension than it does with impact extension. In fact, impact extension involves a notion of constitution that employs the inverse relation: a social institution co-constitutes an engagement with the world as a given cognitive process (by massively extending its impact) not because the institution is part of the engagement, but because the engagement is part of the institution.

If the claim of socially extended cognition is understood in terms of impact extension, the problem of cognitive bloat does not arise. For the claim is no longer that a given social institution is part of a cognitive process, but rather that a cognitive process is part of a social institution.

## Causality, Coordination, and Reciprocal Cognitive Dependency

The notions of causal coupling and functional integration are perfectly at home in the context of implementation extension. The implementation base of a given cognitive process, understood along functionalist lines, consists of causally connected parts that together realize a given functional state or process. Such a base can be extended by causally coupling with further items so that its functionality is increased. This is functional integration. But what about impact extension? As discussed in section “Introduction,” the notion of coupling is used in the context of socially extended cognition as well. If socially extended cognition is an instance of impact extension, this would suggest that impact extension hinges on causal coupling as well. Although I will not deny that impact extension involves causal coupling, my claim is that causal coupling is not the most important principle behind impact extension.

The most important principle behind the cognitive extension offered by institutions—impact extension—is the normativity that comes with the organization and coordination of tasks, roles and actions that is characteristic of an institution.^4^ What extends the impact of putting a scribble on a piece of paper so that it makes me the owner of a house, say, is not just the causal contact of the pen on the paper, nor even the causal contact between the paper and the brain of a notary, a solicitor, a broker, a former owner, or a potential squatter, but the fact that the paper is treated by these as conferring specific rights and obligations that are respected by all. This is a normative practice—a practice in which keeping to specific organized roles is the norm and in which deviation is sanctioned. A legal system is first and foremost a collectively enacted system of coordinated actions. And this coordination is the result of the perceived normativity of the rules governing the system. This abstract description applies to all social institutions that can be said to extend our cognitive abilities. The main differences between legal systems, educational systems, systems of cultural conventions and other “mental institutions,” are in the rules that govern the different systems, connected with the goals of the systems, and in the ways in which deviation from norms is sanctioned (Bicchieri, 2005).

The generally perceived normativity of the rules that govern a given institutional practice—whether enforced or not—allows for the kind of predictability of a given practice that is a precondition for the idea of socially extended cognition. The predictability of the proceedings of a given social institutions is the equivalent of the reliable availability and automatic endorsement of notes in Otto’s notebook. Without sufficiently felt normative force of the principles governing an institutional practice, a practice ceases to be reliable enough to extend the impact of cognitive engagements with the world. If only some people respect the rights conferred to me on the basis of a signed contract, a fading social institution will no longer extend my cognitive engagements and signing a contract will no longer make me a house owner.

The emphasis on normativity, organization and coordination is intended to contrast with the mere mechanical causality that governs artifact-extended cognition. The structures that socially extend our cognitive abilities consist not just of physical artifacts but of (many) other people. We can be causally coupled with other people in many ways, but unless these other people behave in more or less predictable ways, such coupling will not yield cognitive extension. For this we need rules or organizing principles with normative force. In an earlier paper (Slors, 2019). I tried to capture the importance of normativity, organization and coordination and to contrast it with mechanical causality by using a distinction between functional integration (or causal coupling) and what I labeled “task-dependency,” the fact that socially extended cognitive engagements with the world only make sense in the context of a social institution. I argued that socially extended cognition is less characterized by functional integration and more by task-dependency. Gallagher et al. (2019) accept the distinction between functional integration and task-dependency, but are critical of the claim that socially extended cognition is characterized by low functional integration and high task dependency. They argue that:

An attorney, for example, has to make the system work by doing certain things that require material engagement with papers, law books, courtrooms, and many other people. What she does may be defined in terms of specific tasks, but those tasks are accomplished only by engaging with instruments and people, and often in flexible and creative ways. Contracts and written (official) documents are instrumentally functional and, at the same time, they are “pieces” of the legal structure that in some cases predefine or scaffold the roles of individuals. That is, at the same time, they are, from the individual’s perspective, functionally instrumental for extending legal reasoning and, from the systems perspective, constitutive parts of the legal structure (Gallaher et al., 2019, 8).

I believe they are right. The contrast between artifact-extended and socially extended cognition that is the topic of this paper need not hinge on claims about low functional integration in socially extended forms of cognition. The point should simply be that even though material engagement is crucially important (Malafouris, 2013), social engagement is different from mere causal coupling, because it involves other minds, organization, coordination and normativity. In fact, this normativity carries over to the material engagements that (Gallagher et al., 2019) are correct to claim are important parts of social institutions as well. An attorney’s engagement with a law book is subtly but crucially different from Otto’s engagement with his notebook due to its being used in the context of reinforcing norms, rather than merely manipulating information. The normative dimension of the practice of law enters into the attorney’s engagement with the law book, and this is absent in Otto’s interactions with his own notebook.

So, my claim is that socially extended cognition differs from artifact extended cognition because the extending structures in the case of socially extended cognition contain (many) other minds, the required predictability of which can only be due to shared rules and principles that define a given social institution, which are perceived to have normative force. Socially extended cognition adds normativity to the causal coupling with other people and with artifacts that socially extended cognition shares with artifact-extended cognition.

There is another difference between socially extended cognition and artifact-extended cognition that is implied by the above discussion, but not made explicit. Artifact-extended cognition is asymmetrical or non-reciprocal. Otto’s mind is extended by his notebook, not the other way around. The material structuring of actors in 16th century London allowed them to memorize more than ten Shakespeare plays simultaneously and thus extended their minds, but not the other way around. By contrast, socially extended cognition is reciprocal. Social institutions extend our cognitive abilities because we contribute to the practices that define these institutions. By contributing we co-constitute these institutions just like these institutions co-constitute our cognitive abilities (see previous section “Implementation-Extension and Impact-Extension”). And since the cognitive abilities of others are just as well co-constituted by social institutions as ours, we contribute to the cognitive extension of others just as they contribute to ours. Social extension of cognition is reciprocal co-constitution of cognitive abilities.

Given that socially extended cognition is different from artifact-extended cognition—it involves an important normative component, and it is characterized by impact-extension rather than implementation extension, which is reciprocal rather than unidirectional—it may be useful to give it a label of its own. Calling both type of cognition “extended” glosses over important differences. Given the reciprocal cognitive dependency in socially extended cognition, I believe the term “symbiotic cognition” is apt.

## Cognitive Symbiosis, Weak and Strong

Let me summarize the defining features of symbiotic cognition that follow from the above discussion. I will first define what I will label “weak symbiotic cognition,” in abstract terms, briefly comment on the defining features and discuss an example as illustration. I will then argue that it is possible that there are forms of socially extended cognition that do not meet the requirements for symbiotic cognition. Weak symbiotic cognition does not hinge on social institutions. The kind of socially extended cognition referred to by Gallagher, by contrast, exemplified by the examples of signing a contract and voting by raising a hand above, does involve social institutions. This is what I will label “strong” or full-blown symbiotic cognition. It involves a further defining feature that I will discuss and elaborate on at the end of this section.

Weak symbiotic cognition, as I will use the term, is:

(i) a form of *socially* extended cognition,
(ii) that involves *impact extension* rather than implementation extension,
(iii) that involves *normativity* in the interactions between persons on top of causal coupling,
(iv) that involves the *reciprocal* co-constitution of cognitive abilities between persons,
(v) where the social co-*constitution* of cognitive abilities is due to the fact that cognitive processes are shaped as parts of *pre-existing social structures*.

Features (ii–v) are further specifications of (i). As I will argue below, it is defensible to claim that some forms of cognition are socially extended without satisfying (ii–v). Features (ii–v) are strongly interconnected; they highlight different aspects of weak symbiotic cognition, but seem to be a package deal, rather than separate individual necessary conditions.

Feature (ii) has been discussed above. It is important to note that impact extension requires a pre-existing social structure. Without a pre-existing legal system, for example, putting a scribble under a document would not amount to signing a contract and becoming a property-owner.

Feature (iii) follows from the distinction between impact extension and implementation extension discussed above. Initiating cognitive engagements because of their assumed extended impact (say, raising a hand in a vote) anticipates predictable behavior of others in the same social structure. This predictability hinges on the felt normativity of structure-sustaining behavior [see (v)].

Feature (iv) does not imply that reciprocal co-constitution of cognitive abilities is necessarily symmetrical. It may well be that by playing different roles in the same social structure we co-constitute different cognitive abilities in each other.

Feature (v) is deliberately vague about the nature of social structures. The term might refer to social institutions, but this need not be the case. There is structure in human interactions when there are identifiable roles that interact in ways that allow us to discern regularities. The sense in which social structures “pre-exist” before symbiotic cognitive processes can occur is metaphysical, and not necessarily temporal (though in most instances it will be temporal as well): without the context of a social structure, a symbiotic cognitive process cannot exist as such.

Various forms of collective cognitive activity satisfy (i–v), without being instances of the type of cognition Gallagher refers to, i.e., cognition in the context of social institutions. Group-memory is a well-researched case in point. While some researchers argue that memory storage and retrieval by groups is impaired relative to the sum memory abilities of the individual members of a group (Pavitt, 2003), there is considerable research that shows that group-level performance adds to the sum of individual performances (see Theiner et al., 2010, 388–389 for a brief but well-argued overview; see Theiner, 2013 and Arango-Muñoz and Michaelian, 2020 for detailed analyses). Daniel Wegner has probably provided the most famous example of this with his notion of a “transactional memory system,” consisting of two or more individuals who have acquired specific, often implicit routines that allow them to divide and combine cognitive labor efficiently. Thus, long-term married couples are capable of remembering much more together than separately (Wegner, 1986). It is important, in such cases, that we do not disrupt the ingrained routines. Assigning a different, new division of cognitive labor, for example, reduces the collective memory capacity of couples demonstrably (Wegner et al., 1991). These routines are instances of the pre-existing social structures referred to in (v).

In general, task division in couples that live together for some time often rigidifies into shared routines, that are usually based on tacit knowledge of individual proclivities and talents, and that usually amount to the automatic complementing of each other’s cognitive efforts. Such routines would make the couple into a symbiotic cognitive systems in terms of the above definition. Let me take the following, simplified case as an example: when on vacation, my wife always takes care of train- plane- or boat-tickets and the planning of when we should go where and what to see, whereas I do navigation and hotel arrangements, tents (in which case my wife determines the campsite) and guesthouses.

This (simplified) arrangement satisfies (ii–v):

(ii) My actions of arranging tent-gear and navigating result in having a complete vacation, including interesting trips, a nice campsite, a boat trip, etcetera, because they are done in the context of a (weakly) symbiotic system. This is a form of impact extension; outside of this context the same actions would not have that effect.
(iii) There is most certainly a kind of normativity involved in our division of cognitive labor. This is based on precedent and on shared assessment of talents which leads to mutual expectations.
(iv) We co-constitute each other’s cognitive abilities. By dividing complementary cognitive tasks and by using many automatized interaction routines that let us share information when necessary (and not when not necessary), we co-constitute each other’s ability to realize a full vacation with roughly half the effort.
(v) These routines—our implicit knowledge of the way in which we divide cognitive labor and share results when necessary—counts as social structure of the relevant kind (i.e., supporting reciprocal co-constitution of cognitive abilities).

Are there forms of socially extended cognition that do not satisfy (ii–v)? I believe that that is possible, depending on how widely we apply the term “socially extended.” For example, the relation between a student and a teacher might be described as socially extended cognition—the student’s cognition is extended by the teacher’s (note that nothing in this paper hinges on calling this an instance of extended cognition). Likewise, a reader’s cognitive abilities might be thought of as being extended by the cognitive activities of a writer. There are reasons to be cautious here in describing such cases as instances of socially extended cognition,^5^ but even if we disregard these, such cases are not instances of symbiotic cognition. For first, and most importantly, these relations do not satisfy (iv): the cognitive extension is a one-way affair and not reciprocal—teachers extend the cognition of students, but not vice versa and writers extend the cognitive abilities of readers, but not vice versa. This might be argued to affect (ii), (iii), and (v) as well. To start with (v), the social structures involved are not structures of the right kind because they do not involve mutual dependency. Also, these relations do not involve the right kind of normativity. There may certainly be normativity involved in these relations or in playing the relevant roles involved, but not necessarily normativity of the kind that renders the behavior of others predictable so that cognitive engagements by the agent are impact-extended. Which means that (ii) is not satisfied either. Having said that, though, nothing hinges on these assessments of the applicability of (ii), (iii), and (v); the non-applicability of (iv) suffices to rule out these cases as cases of symbiotic cognition.^6^

I have labeled forms of symbiotic cognition that do not involve social institutions “weak symbiotic cognition,” because they differ in one important respect from socially extended cognition of the type Gallagher discusses. I believe that the discussion of the previous sections suffices to show that (i–v) apply to Gallagher’s cases. But these cases have a striking feature that is lacking in the case of a married couple jointly planning and having a vacation or the case of collective memory. The cognitive engagements Gallagher discusses are only intelligible *within* the context of their respective institutions. Many of our daily cognitive activities have this property. Signing a contract is not intelligible in abstraction from a legal system, voting is not intelligible in abstraction from a social structure which allows for joint decision making, being polite by shaking hands is not intelligible in abstraction from a system of cultural conventions, etcetera. What I will label “strong” or full-fledged symbiotic cognition, then, adds one more requirement to (i–v):

(vi) Cognitive processes are *possible* and *intelligible* only within the context of a social institution.

Crucially, the example of married couples with ingrained automatized routines, or transactional memory systems, are not examples of strong symbiotic cognition. For the individual cognitive processes within such symbiotic systems *are* intelligible in abstraction from the system. My activity of navigating or booking a hotel does not require my wife’s activity of planning trips and booking tickets to be intelligible. Neither does the individual memory-contribution of an individual to a transactive memory system require reference to other people to be intelligible as a memory process.^7^ Weak symbiotic cognitive systems combine individual cognitive processes, that do not require the system to exist, into a larger system that is beneficial to participants. Strong symbiotic cognition, by contrast, cannot be reduced to a collection of individually intelligible cognitive processes. It is only in connection with the whole system that strongly symbiotic cognitive processes are cognitive processes at all. It is not just that the whole is more than the sum of its parts (see footnote 7), the point is rather that there are no identifiable relevant parts without the notion of the whole.

Take the case of signing a contract again. What it *means* to sign a contract involves reference to a very complex social structure in which rights and obligations exist and can be changed. “Rights and obligations” refers to very specific norm-guided, socially structured behavior. It is not possible to identify that behavior fully, in turn, without referring back to contracts. The roles and regularities of the social structures involved in strong symbiotic cognition are *holistically* inter-defined (Slors, 2019). To define the role of a barrister, one has to refer to the rule of law, and to roles of citizens, judges, clerks and many others. And to define these other roles, reference to the roles of barristers will have to be made. To define the role of a board member, one has to refer to the whole organizational structure of a company.

For the type of roles and regularities to exist that can and need to be holistically inter-defined, a certain degree of complexity is required. Strongly symbiotic systems, then, are likely to be much larger systems than transactional memory systems. A legal system, typically, is enacted by a whole society. A company is enacted by a very large group of people, and can exist only within an economic arrangement that involves whole countries. Strong symbiotic cognition, then, is not just a more stringent sub-variety of weak symbiotic cognition.

The holistic inter-defining of roles and regularities implies that strongly symbiotic cognitive engagements or processes are necessarily aimed at accomplishing a given state of affairs *within* the relevant symbiotic system. Any cognitive engagement that counts as executing a system-defined role implies the involvement of other people playing their respective roles in that same system. Signing a contract is what it is because it affects the roles, obligations and rights of other people (a former house owner, say, can no longer determine what is to be done with a house once it is yours, due to you signing a contract; she can no longer determine this *as* a citizen who falls within the same legal system as you do). Shaking hands as a greeting opens up a new space of social interaction possibilities due to the fact that those involved all participate in the same system of cultural conventions—it is a “move” *within* the “game” of social etiquette that is meaningless or weird to anyone who does not share your conventions.

Feature (vi), then, transforms weak symbiotic cognition into a qualitatively different kind of cognition. If (vi) is added to (ii–v), and the five features together are taken as interconnected, then (ii–v) are substantially strengthened. Of course feature (v) is further defined by limiting the pre-existing social structures to social institutions. But this affects the other features too. Impact extension (ii) within a strongly symbiotic system is substantially more encompassing than impact extension in a weakly symbiotic system. Setting a whole reorganization of a company in motion by raising a single hand illustrates the point. This is a different scale of impact-extension than having a whole vacation with half the work. (iii) The normativity involved in social institutions is not merely dependent on precedent and implicit assessment of talents and proclivities. Precisely because it applies to much larger groups, it is usually reinforced, either explicitly, as in legal systems, or implicitly, as in a system of social etiquette. (iv) The co-constitution of cognitive abilities in strongly symbiotic systems is much more elaborate than in weakly symbiotic systems. First of all this is because many more people are involved. But secondly this is because most social institutions, instill a wide range of “new” cognitive abilities in those who help to enact them.

As said, I take Gallagher to refer to strong of full-fledged symbiotic cognition in his discussion of socially extended cognition. In the remainder of this paper I will refer to this type of cognition simply as “symbiotic cognition.”

## Symbiotic Cognition, Cognitive Integration and Distributed Cognition

So far, I have limited the discussion to the literature on extended cognition, arguing that symbiotic cognition differs from “normal,” artifact-extended cognition in some important respects. There are other theories about the essential embeddedness of our cognitive systems. Richard Menary’s notion of cognitive integration does emphasize the expansion of our cognitive repertoire by engaging with a wide variety of cultural items, including social structures, but without making claims about the extension of our cognitive systems as such. Edwin Hutchins’ notion of socially distributed cognition, by contrast, allows for whole social institutions to count as cognitive systems. I have argued that the literature on extended cognition has swept an important distinction under the carpet; it has not sufficiently recognized that socially extended cognition is—at least very often—a type of cognition of its own, fundamentally different from artifact-extended cognition. But it may well be that this distinction is respected by the notions of integrated cognition or distributed cognition. I which case I may have said nothing new. I will briefly argue, however, that neither cognitive integration, nor distributed cognition is very sensitive to the distinction I have argued for above.

The idea of cognitive integration is in many respects very close to the idea of extended cognition. Cognitive integration is also close to the enactivist view in that it emphasizes that cognition consists of bodily manipulations of the world, often involving man-made cognitive devices (alternatively, it may, according to Menary, also consist of mental simulations of such manipulations). Cognitive processes are cognitive practices, and these can be hugely expanded by involving a host of different items. The items mentioned in the cognitive integration literature fall in the same (wide) range as the devices referred to by extended cognition theorists. The crucial difference with extended cognition is that while according to Menary items such as linguistic symbols, smart phones, abacuses and social institutions allow for a whole new range of cognitive practices, they are enabling conditions for such practices, rather parts of our minds. In this respect, Menary is closer to those who argue that external devices scaffold our cognition, rather than extend it (e.g., Sterelny, 2010).

It should be noted that the notion of cognitive extension that Menary rejects is a variant of what I have labeled “implementation extension” above. Even though he tends toward an enactivist notion of cognition rather than a classical functionalist one, he still speaks of cognition “supervening” on a realization base and thinks of cognitive extension in terms of enlarging this base. This raises the question whether perhaps impact-extension might be compatible with the idea of cognitive integration. The similarity between the enactivist notion of cognitive engagement and Menary’s notion of cognitive practices might suggest this. Indeed, there are clear similarities. Menary speaks of the “transformation” of our minds by cognitive artifacts and our interactions with them in a way that suggests that manipulating these artifacts has a cognitive yield in the context of cognitive practices that the same manipulation would not have outside of such practices. The impact of an ignorant infant who happens to manipulate numeric symbols such that they accidentally represent a calculation differs from the impact of a mathematically trained person who performs the same manipulation. This is akin to the difference between someone coincidentally putting a scribble on a piece of paper and someone signing a real contract. The practice extends the impact of the manipulation.

However, even though it may be argued that this type of “transformation” of cognitive processes is very much like impact-extension, this does not mean that the idea of cognitive integration already contains or implies the notion of symbiotic cognition. On Menary’s view, all cognitive integration is somewhat like impact-extension. The contrast between socially extended/integrated and artifact-extended/integrated cognition—or between what I would prefer to call extended and symbiotic cognition—is not made. Hence, in this respect it will not help to abandon extended-cognition talk in favor of cognitive integration.

What about socially distributed cognition? On Hutchins’ original proposal, (Hutchins, 1995) socially distributed cognition is a view on cognition that is much like the idea of group minds (Theiner et al., 2010). The point of this view is that it is perfectly possible for a group of people to jointly carry out certain cognitive tasks. Can social institutions be viewed as cognitive systems? On the wide characterization of “cognitive system” employed by Hutchins, 2014 in his later work (e.g., 2014), they can. For here the criterion is not that a system has a given task (as in Hutchins earlier work), but that it consists of integrated cognitive elements such that i.e., multiple human beings in conjunction with a cultural niche replete with cognitive artifacts counts as such a system. Hutchins speaks of “a cognitive ecosystem.” A social institution can certainly be viewed as a cognitive ecosystem. Cognition in a cognitive ecosystem is not implementation-extended, but impact-extended. Like symbiotic cognition, and unlike extended cognition, Hutchins emphasizes that distributed cognitive systems have no center—there is no one brain that is extended by others, but there is what I called reciprocal extension.

In many respects, therefore, symbiotic cognition can be viewed as a variant of the cognitive ecosystems view implied by later versions of the idea of distributed cognition. The one thing that is missing, however, like in the case of cognitive integration, is the relevant contrast between extended and symbiotic cognition. Hutchins (2014, 36–38) still thinks of extended cognition as a possible variant of distributed cognition. Thus, he ignores the difference between causal coupling and reciprocal social-normative coupling that involves organization and action coordination. To sum up, then: some elements of symbiotic cognition can be found in the ideas of integrated and distributed cognition, but the relevant contrast between symbiotic and extended cognition that I have been arguing for in this paper is still absent.

## Conclusion

I have argued that there is an important distinction between cognitive extension as the extension of the causal-functional implementation base of cognitive processes, which is best applicable in cases where cognition is extended by physical artifacts only, and cognitive extension as the idea that our cognitive engagements with the world have massively enhanced impact in the context of normative, rule-based coordination of actions in a social practice. Though both types of cognition might equally well be called “extended,” they are extended in radically different ways. In order to mark this difference, and given the reciprocal cognitive co-constitution between humans in impact-extended cognition, I have proposed to label what is now known as socially extended cognition “symbiotic cognition.”

## Author Contributions

The author confirms being the sole contributor of this work and has approved it for publication.

## Conflict of Interest

The author declares that the research was conducted in the absence of any commercial or financial relationships that could be construed as a potential conflict of interest.

## Footnotes

1 A second reason to leave this discussion for what it is, is because the arguments for brain chauvinism are directed against what I will later call “implementation extension.” The notion of “impact extension” which I will argue characterizes socially extended cognition is not directly susceptible, I believe, to Adams and Aizawa’s critique.

2 I am using scare quotes because I am not implying any commitment to mechanistic explanation in cognitive science. I will argue in this section that an enactivist view on cognition yields a different notion of cognitive extension than a functionalist view. My formulation must therefore be enactivist-friendly. Although I do not believe that mechanistic explanation and enactivism are enemies (Abramova and Slors, 2019), many enactivists do not accept a mechanistic style of explanation in cognitive science. By “mechanism” I mean something like processes that are responsible for the way a cognitive system functions.

3 One could say that these social practices/institutions allow a person to engage with a much bigger portion of the world. Thus, instead of impact extension we could also speak of “engagement extension.” For the sake of simplicity I will use one label—impact-extension—only.

4 There are other kinds of normativity. In particular, there are norms for the manipulation of cognitive devices that I would count as instances of artifact-extended cognition. Menary (2010, 238–241) gives an instructive overview of these. As Menary himself emphasizes, however, such cognitive normativity should be distinguished from social normativity. The contrast I wish to make between causal and normative connections pertains to social normativity.

5 One problem here is that while there is no impact-extension involved, it would be somewhat odd to speak of implementation-intention, unless we want to include other people in the implementation base of one’ own mental processes.

6 The point that is made here about socially extended cognition can also be made about affective social scaffolding. Stephan and Walter (2020, section 4), mention examples such as seeing a psychotherapist, confessing to a priest, the emotion regulation involved in infant-caregiver interactions (see also Krueger, 2013), and the transformative effect of social media on our affective mindset (or our mindset in general). However, in all but the last of these examples, the reciprocity that is characteristic of symbiotic cognition is absent or so much diminished that I would consider them borderline cases at best.

7 This is not to say that collective memory cannot be an emergent process. It can. Emergence hinges on the way that an overall process such as collective remembering depends, ontologically, on its constituent processes (see Arango-Muñoz and Michaelian, forthcoming, sections 11.3.2 and 11.4 for a discussion of different forms of emergence in the context of collective memory). It does not require that the constituent processes be definable or intelligible only in ways that refer to the overall process whose emergence they contribute to.
